# Genome-Wide Association Study Identifies Variants in PMS1 Associated with Serum Ferritin in a Chinese Population

**DOI:** 10.1371/journal.pone.0105844

**Published:** 2014-08-27

**Authors:** Ming Liao, Jianying Shi, Lirong Huang, Yong Gao, Aihua Tan, Chunlei Wu, Zheng Lu, Xiaobo Yang, Shijun Zhang, Yanlin Hu, Xue Qin, Jianling Li, Gang Chen, Jianfeng Xu, Zengnan Mo, Haiying Zhang

**Affiliations:** 1 Institute of Urology and Nephrology, First Affiliated Hospital of Guangxi Medical University, Nanning, Guangxi, PR China; 2 Center for Genomic and Personalized Medicine, Guangxi Medical University, Nanning, Guangxi, PR China; 3 School of Public Health, Guangxi Medical University, Nanning, Guangxi, PR China; 4 General Practice School, Guangxi Medical University, Nanning, Guangxi, China; 5 Department of Pharmacology, Guangxi Medical University, Nanning, Guangxi, PR China; 6 Medical Scientific Research Center, Guangxi Medical University, Nanning, Guangxi, PR China; 7 Department of Clinical Laboratory, First Affiliated Hospital of Guangxi Medical University, Nanning, Guangxi, PR China; 8 Institute of Cardiovascular Disease, First Affiliated Hospital of Guangxi Medical University, Nanning, Guangxi, PR China; 9 Pathology Department, First Affiliated Hospital of Guangxi Medical University, Nanning, Guangxi, PR China; 10 Center for Cancer Genomics, Wake Forest University, School of Medicine, Winston-Salem, North Carolina, United States of America; 11 Center for Genetic Epidemiology, Van Andel Research Institute, Grand Rapids, Michigan, United States of America; 12 Urology Department, First Affiliated Hospital of Xinxiang Medical College, Xinxiang, Henan Province, China; MOE Key Laboratory of Environment and Health, School of Public Health, Tongji Medical College, Huazhong University of Science and Technology, China

## Abstract

Only a small proportion of genetic variation in serum ferritin has been explained by variant genetic studies, and genome-wide association study (GWAS) for serum ferritin has not been investigated widely in Chinese population. We aimed at exploring the novel genetic susceptibility to serum ferritin, and performed this two stage GWAS in a healthy Chinese population of 3,495 men aged 20–69 y, including 1,999 unrelated subjects in the first stage and 1,496 independent individuals in the second stage. Serum ferritin was measured with electrochemiluminescence immunoassay, and DNA samples were collected for genotyping. A total of 1,940,243 SNPs were tested by using multivariate linear regression analysis. After adjusting for population stratification, age and BMI, the rs5742933 located in the 5′UTR region of *PMS1* gene on chromosome 2 was the most significantly associated with ferritin concentrations (*P*-combined = 2.329×10^−10^) (β = −0.11, 95% CI: −0.14, −0.07). Moreover, this marker was about 200kb away from the candidate gene *SLC40A1* which is responsible for iron export. *PMS1* gene was the novel genetic susceptibility to serum ferritin in Chinese males and its relation to *SLC40A1* needs further study.

## Introduction

Ferritin, one of the key proteins regulating iron homeostasis, is a widely available clinical indicator to evaluate iron status [1]. As a serum marker of iron status, elevated serum ferritin is not only used to screen for iron overload, but also applied to predict metabolic syndrome and type 2 diabetes [2]–[5]. Growing evidences suggested that genetic factors contributed 20–30% of the variation to blood iron concentrations [6]–[9]. However, only a small proportion of genetic variation in serum ferritin has been explained by variant genetic studies [10]. It was well known that the two subunits of ferritin were synthesized under the control of different genes in chromosomes 11 and 19, respectively [11], [12]. As most cases of genetic hemochromatosis were associated with the C282Y (a cystine to tyrosine mutation at position 282) [10], Milet *et al* reported firstly that *BMP2* gene was associated with serum ferritin in C282Y homozygotes patients [13]. Later, it was demonstrated that *TMPRSS6* polymorphisms were significantly associated with ferritin as well [14].

The genome-wide association study (GWAS) is a powerful and unbiased tool for the identification of common genetic variants associated with complex traits. To the best of our knowledge, there have been two GWASs for serum ferritin, both of which were performed on Australian samples [10], [15]. In the first GWAS on adult female monozygotic twins, genes *TF* and *HFE* were associated with serum ferritin [10], while the other GWAS on adolescent and adult individuals from twin families reported the association of serum ferritin with gene *TMPRSS6* [15]. Moreover, in a meta-analysis of two GWASs in both semi-isolated and outbred populations from Italy and the USA, the *HFE* locus has been identified to be associated with serum ferritin, and the involvement of *TF*, *TMPRSS6* and *HFE* genes in the maintenance of iron homeostasis was confirmed [16]. Recently, in a candidate gene study of Chinese Hans, two variants of *TMPRSS6* gene (V736A and D521D) were confirmed to be associated with ferritin concentrations, but the *TF* and *HFE* genes were not studied [14]. Considering that only a small proportion of genetic variation has been identified for serum ferritin, and there were diversities in allele and genotype frequencies in different ethnic populations, it is necessary to explore common genetic variants associated with the serum ferritin in Chinese. Thus, we conducted this two-stage GWAS in a healthy Chinese male population in search of population-specific genetic variations associated with serum ferritin.

## Methods

### Study population

A two-stage GWAS was performed to identify the genes/loci that influence serum ferritin concentrations.

Stage 1 of the GWAS included 1,999 unrelated healthy Chinese men aged 20–69 years old from the Fangchenggang Area Male Health and Examination Survey (FAMHES). The FAMHES is described elsewhere [17]. Briefly, it was designed to investigate the effects of environmental and genetic factors. All subjects were free of stroke, primary hypertension, diabetes mellitus, rheumatoid arthritis, hyperthyroidism, tumors, coronary heart disease, and hepatic or renal dysfunction. All men who participated in physical examinations in the Medical Centre of Fangchenggang First People’s Hospital from September 2009 to December 2009 were invited to participate in the study (n = 4,364). A total of 4,303 participants (98.6%) provided informed consent and blood samples. There were 2,012 people randomly selected from southern Chinese Han ethnicity. After selected by age criteria, a total of 1,999 individuals passed the call rate of 95% and were used in the final statistical analysis.

Stage 2 of the GWAS consisted of 1,496 healthy Chinese men aged 20–69 years old. They were randomly selected from male participants who participated in physical examinations from September 2009 to September 2010 in the Medical Centre of Fangchenggang First People’s Hospital, Guigang People’s Hospital and Yulin First People’s Hospital. Stage 2 samples from Fangchenggang First People’s Hospital were independently recruited from the stage 1 samples. In stage 2,996 were of Han ethnicity and 500 were of Zhuang ethnicity. The same recruitment strategy was used in stages 1 and 2.

Comprehensive health information was collected through clinical examination, and additional demographic information was obtained via a standardized questionnaire. We obtained written documentation of informed consent from all study participants, and the research protocol was approved by the local ethics committee. Both smoking and drinking in two stages were assessed on the basis of a self-administered life-style questionnaire according to the same protocol. Respondents that reported smoking currently (daily smoking >6 months) were coded as smokers, and those reported drinking any beverage ‘more than once a year’ were coded as drinkers, whereas others were non-drinkers [18]. The study was approved by the Ethics and Human Subject Committee of Guangxi Medical University,

### Measurement of serum ferritin

The description of the laboratory test has been previously reported in detail [19]. Briefly, about 10 ml overnight fasting venous blood specimens were collected between 8∶00 and 11∶00 am and were transported frozen to the testing center of Department of Clinical Laboratory at the First Affiliated Hospital of Guangxi Medical University in Nanning in two hours, which were centrifuged within 15 to 25 min and stored at −80°C until analysis. Ferritin was measured with electrochemiluminescence immunoassay on COBAS 6000 system E601 (Elecsys module) immunoassay analyzer (Roche Diagnostics, GmbH, Mannheim, Germany) with the same batch of reagents, and the inter-assay coefficient of variation was 3.4%.

### Genotyping

In our study, two different platforms were used for single nucleotide polymorphism (SNP) genotyping. For stage 1, genotyping was performed by using the Illumina Omni 1 platform. The Sequenom iPLEX system (Sequenom, Inc., San Diego, CA, USA) was used in stage 2. Polymerase chain reaction and extension primers were designed using Mass ARRAY Assay Design 3.1 software (Sequenom, Inc.). Manufacture’s iPLEX Application Guide (Sequenom, Inc.) was performed for genotyping procedures. All of the genotyping reactions were performed in 384-well plates. Each plate included a duplicate for three or four participants selected at random, as well as six to nine negative controls in which water was substituted for DNA. The average concordance rate was 99.8%.

### Statistical analysis

Quality control procedures were applied to 1,999 unrelated individuals that were genotyped using the Illumina Omni-Express platform [17]. Total 1,999 samples passed the call rate of 95% and were included in the final GWAS analysis. We then applied the following QC criteria to filter SNPs: *P*<0.001 for the Hardy–Weinberg equilibrium test, minor allele frequency <0.01 and genotype call rate <95%. Based on these criteria, 709,211 SNPs were retained. The IMPUTE computer program [20] was then used to infer the genotypes of SNPs (e.g. SNPs catalogued in Hapmap Phase II CHB population release #24) in the genome that was not directly genotyped. A posterior probability of >0.90 was applied to call genotypes that were imputed using IMPUTE software. After applying the same QC criteria, as used above, a total of 1,940,243 SNPs remained in the final analysis. Analysis for ferritin was performed on log-transformed values. Linear regression implemented in PLINK [21] was used to estimate the SNP association under the assumption of an additive relationship between the number of copies and the residual log-transformed ferritin value. Population stratification was estimated by a principal component approach, as implemented by EIGENSTRAT software [22] Clinical covariates utilized in the linear regression modeling included age at the time of ferritin measurement, body mass index (BMI, weight in kg divided by the height in m^2^). For regions with multiple SNPs that were significant at *P*<10^−6^, multivariate linear regression analysis was applied to test the independence of the respective SNPs. Only the SNPs that remained significant at 10^−6^ in the multivariate analysis were selected. The combined analysis of two-stage data was performed using a linear regression, adjusting for the covariates (population stratification, age, BMI and stage information). The β coefficient and 95% confidence internal (95% CI) was reported.

## Results

**Figure 1:**
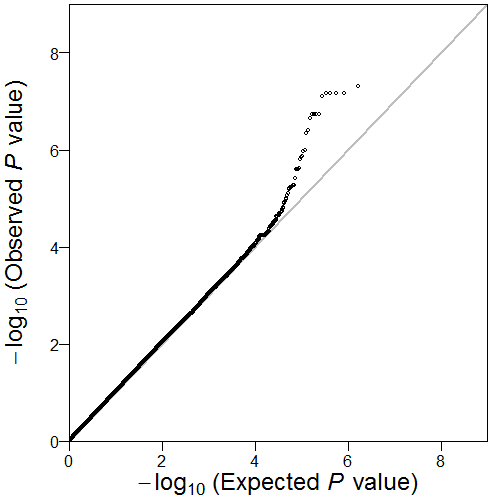
Quantile-Quantile plot of genome-wide quantitative trait loci mapping for log-transformed serum ferritin concentrations.

**Figure 2:**
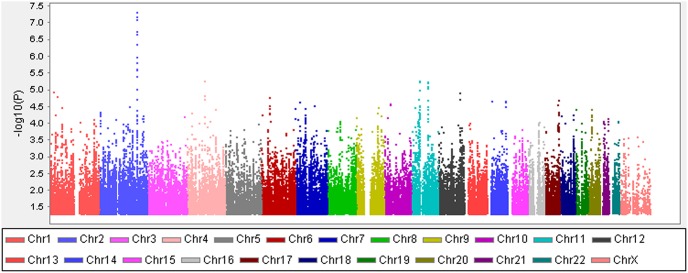
Manhattan plot of genome-wide association analysis for serum ferritin concentrations. X-axis shows chromosomal positions. Y-axis shows –log10 P-values from the linear regression adjusting for population stratification, age and BMI and stage information. Candidate gene names are showed for the significantly associated loci.

The general characteristics of the samples in this study were described in **Table 1**. There were 1,999 participants in stage 1 and 1,496 participants in stage 2. No significant difference was observed between the two stages in age distribution (37.5 versus 37.3 years, *P* = 0.54), BMI (23.3 versus 23.5 kg/m^2^, *P* = 0.18) and smoking behavior (*P* = 0.66), excepting for alcohol consumption (*P* = 0.02). As showed in **Figure 1**, the Quantile-Quantile plot of adjusted P values with the inflation factor of 1.01 show no systematic bias. When the top two Eigens were added to other covariates in the GWAS analysis, similar results were obtained. The inflation factor indicated that there was no population substructure in the GWAS analysis. The genome-wide association results were presented in the Manhattan plot (**Figure 2**).

**Table 1:** General characteristics of participants in the two-stage GWAS.

Characteristics	First stage	Second stage	P-value^a^
n	1,999	1,496	
Age, years(mean±SD)	37.5±11.1	37.3±10.8	0.54
Smoking, n(%)			0.66
Yes	1015(50.8)	771(51.5)	
No	984(49.2)	725(48.5)	
Alcohol drinking, n(%)			0.02
Yes	1704(85.5)	1165(82.6)	
No	288(14.5)	246(17.4)	
Body mass index (kg/m^2^)	23.3±3.44	23.5±3.34	0.18

a T-test was used to compare means of the continuous variables between the first and the second stage; Chi-square test was used to compare the differences for categorical variables.

In stage 1, we performed the multivariate regression analysis adjusting for population stratification, age, BMI, and selected the SNPs with *P*<1.0×10^−6^. As showed in **Table 2**, we totally identified nine loci on chromosomes 2. The rs5742933, located in the end of 5′UTR region of gene postmeiotic segregation increased 1 (*PMS1*) was the most significantly associated with ferritin concentrations (*P* = 4.699×10^−8^) (β = −0.05, 95% CI: −0.07, −0.03). It was also in strong linkage disequilibrium (LD) with the other eight SNPs (all both R^2^ and D’>0.9), not only located in *PMS1* but also in *ANKAR, OSGEPL1* and *ORMDL1.* According to LD and hap-block of SNPs, only rs5742933 was selected to be further confirmed.

**Table 2:** SNPs associated with serum ferritin concentrations.

									Mean (ng/ml)					
SNP	Chromosome	BP^a^		Gene	Hwe	Allele	Allele(m)^b^	MAF^b^	mm^c^	Mm^c^	MM^c^	R^2^	D’	P^c^
rs5742933	2q31.1	190,357,561		*PMS1*	0.07	C/G	C	0.24	296.5	347.5	388.2	1.000	1.000	4.70×10^−8^
rs3791770	2q31.1	190,382,080		*PMS1*	0.04	T/C	T	0.23	306.4	348.5	388.1	0.997	0.991	3.75×10^−6^
rs3791773	2q31.1	190,390,877		*PMS1*	0.10	C/G	C	0.24	298.8	348.4	388.1	0.997	0.991	3.68×10^−7^
rs1550388	2q32.2	190,312,025		*ANKAR*	0.14	T/C	T	0.23	301.9	346.5	387.9	0.962	0.989	1.33×10^−7^
rs1225101	2q32.2	190,313,666		*ANKAR*	0.14	A/T	A	0.23	301.9	346.5	387.9	0.962	0.989	1.33×10^−7^
rs1898560	2q32.2	190,320,572		*OSGEPL1*	0.09	C/A	C	0.24	295.9	347.8	388.0	0.997	0.991	5.44×10^−8^
rs4666783	2q32.2	190,321,225		*OSGEPL1*	0.09	T/C	T	0.24	295.9	347.8	388.0	0.997	0.991	5.44×10^−8^
rs2289404	2q32	190,344,259		*ORMDL1*	0.09	A/G	A	0.24	295.9	347.9	388.1	0.994	0.995	6.15×10^−8^
rs3791767	2q32	190,348,160		*ORMDL1*	0.09	A/G	A	0.24	295.9	347.8	388.0	0.997	0.991	5.44×10^−8^

a Genomic position is based on NCBI build 36.

b m, minor allele, M, major allele, MAF indicates the minor allele frequency for allele m; MM indicates serum ferritin concentrations for homozygous carriers of major alleles, Mm indicates heterozygous carriers and mm indicates homozygous carriers of minor alleles.

c P-values were calculated based on multivariate linear regression analysis adjusted for population stratification, age and BMI assuming an additive model.

**Figure 3:**
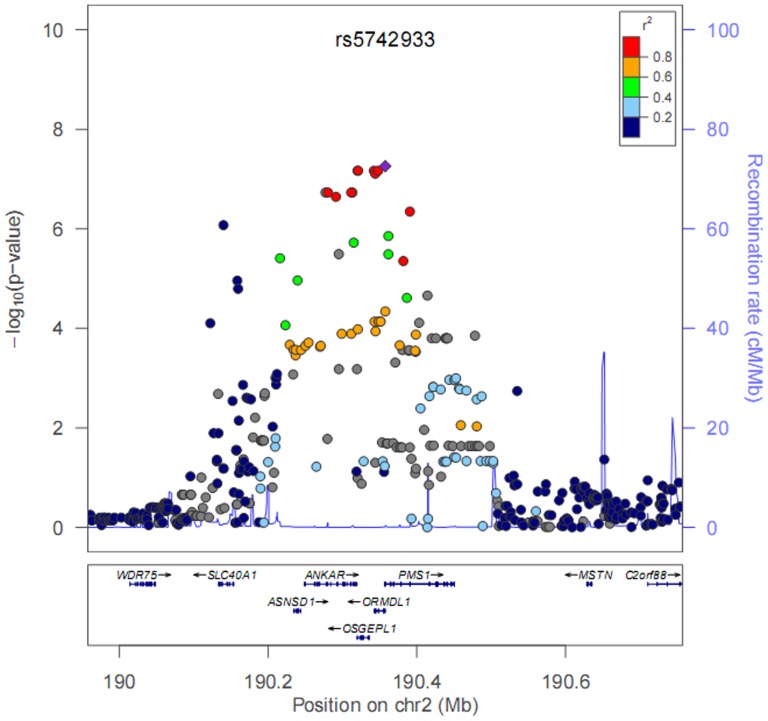
Association of serum ferritin concentrations with SNPs at chromosome 2. X-axis shows base positions from 190,414 Kb to 190,357 Kb. Y-axis shows –log10 P-values from linear regression adjusting for population stratification, age and BMI and stage information. Ferritin concentrations were log-transformed and fit for a normal distribution. The bottom panels describe all genes in the region.

In stage 2, the association between rs5742933 and serum ferritin was validated by examining 1,496 healthy subjects. The rs5742933 reached with a *P*-value of 6.777×10^−4^ (β = −0.09, 95% CI: −0.14, −0.04) in the second stage after adjusting for the same covariates. It remained significantly associated with ferritin concentrations after further adjusting for the stage information (combined-*P* = 2.329×10^−10^) (β = −0.11, 95% CI: −0.14, −0.07). Interestingly, rs5742933 was about 200 kb away from the candidate gene *SLC40A1* as showed in **Figure 3**. The genotypes of rs5742933 showed significant association with ferritin (*P*<0.001); serum ferritin basically decreased with increasing numbers of minor allele C (**Table 3**). However, the genotypes of rs5742933 did not show statistically significant association with smoking or alcohol consumption (both *P*>0.05).

**Table 3:** General characteristics of participants in the two-stage GWAS stratified by the rs5742933.

	First Stage				Second Stage			
	CC	CG	GG	P-value^b-^	CC	CG	GG	P-value^b^
Age, years(mean±SD)	38.5±11.1	37.6±11.2	37.3±11	0.55	37.7±12.6	36.5±10	37.4±10.7	0.31
Body mass index,(kg/m^2)^	23.6±3.1	23.1±3.3	23.4±3.4	0.19	22.4±4.1	22.7±5.5	22.7±5.5	0.85
Ferritin, (ng/ml)^a^	248.9±1.9	292.1±1.9	328.9±1.8	<0.001	208.2±1.9	214.8±8.2	242.7±6.2	<0.001
Smoking, n(%)				0.08				0.71
Yes	43(43.4)	365(48.9)	607(52.7)		31(56.4)	264(52.1)	434(50.9)	
NO	56(56.6)	382(51.1)	544(47.3)		24(43.6)	243(47.9)	418(49.1)	
Alcohol drinking, n(%)				0.18				0.27
Yes	80(80.8)	631(84.5)	996(86.5)		31(57.4)	345(68.3)	569(67.3)	
NO	19(19.2)	116(15.5)	155(13.5)		23(42.6)	160(31.7)	276(32.7)	

a Ferritin levels were log-transformed and the values presented were back-transformed.

b One-way ANOVA was used to compare means of the continuous variables, while chi-square test was used to compare the differences for categorical variables in subgroups stratified by rs5742933.

## Discussion

Serum ferritin plays an important role in clinical researches; however, comprehensive genetic assessments of the variability in ferritin remain poorly studied. We performed this two-stage GWAS in 3,495 Chinese adult males in search of the genetic variations associated with serum ferritin. We found that the rs5742933 located in the 5′UTR region of *PMS1* gene was the most significantl SNP associated with serum ferritin, which have never been reported before in any population.

The rs5742933 is in the 5′UTR of *PMS1* gene that is a DNA mismatch repair gene. This SNP was predicted as an exonic splicing silencer that may inhibit or silence splicing of the pre-mRNA, or as a transcription factor binding site that may affect the level, location, or timing of gene expression [23]. As one of the potentially functional polymorphisms in *PMS1*, the rs5742933 may serve as candidate prognostic marker of clinical outcome of non-small-cell lung cancer [24]. PMS1 was expressed in many tissues including haematopoietic cells [25]. In a study of Japanese children with aplastic anemia (AA), IgG antibodies against PMS1 were detected in 10% patients (3/30), while no antibody responses to PMS1 in normal volunteers [26]. In another multicentre study of more children with AA, PMS1 antibodies were deselected in 14.6% (15/103) of patients, nevertheless, but the role of PMS1 antibodies in AA has not yet been determined [27]. Besides, the *PMS1* gene was also associated with systolic blood pressure and hypertension in a GWAS of 29,136 participants from six large prospective observational studies in the CHARGE Consortium [28]. Although no previous studies examined the relationship between *PMS1* and ferritin, our finding may indicate the involvement of DNA mismatch repair in genetic control of ferritin concentrations.

Interestingly, the *PMS1* gene was near *Ferroportin 1* (*SLC40A1*), which is also known as *HFE4* or *SLC11A3*. The protein encoded by this gene is a cell membrane protein that may be involved in iron export from duodenal epithelial cells. Defects in this gene are a cause of hemochromatosis type 4. The A77D mutation and the Val162 deletion in the gene *SLC40A1* would lead to hyperferritinaemia and reticuloendothelial iron overload [29]. The *SLC40A1* gene silencing in human macrophages would induce iron retention and enhance ferritin synthesis [30]. Moreover, the *SLC40A1* gene may interact with the *HFE, TFR2* genes, causing hyperferritinemia and an iron overload phenotype [31]. In the present study, the most significant SNP was located in the 5′UTR of *PMS1* and in strong LD with SNPs covering *PMS1* (both R^2^ and D’>0.99), but in considerably weak LD with SNPs covering *SLC40A1* (both R^2^ and D’<0.20). These results suggested that *PMS1*, rather than *SLC40A*, was associated with serum ferritin, although these two genes were much closed. Nevertheless, we can not rule out the possibility of epigenetic effect between *PMS1* and *SLC40A1* in the genetic control of serum ferritin.

Although we found the *PMS1* was significantly related with the serum ferritin concentrations in Chinese males, we failed to report the other loci previously identified in other populations, such as *HFE*, *BMP2*, and *TMPRSS6*. Firstly, *HFE* was associated with serum ferritin in the GWAS for adult female monozygotic twins from Australia [10], consistent with the previous study [32], [33]; however, our study failed to confirm this. According to HapMap data, the C282Y and H63D mutation of *HFE* gene is common in Europeans but rare in Chinese. This characteristic of *HFE* may partly explained our negative results. Secondly, the *BMP2* was associated with ferritin in the previous GWAS for elderly Chinese women with iron-deficiency anemia [34]. We failed to replicate the same result probably due to the difference in serum ferritin levels between elder females with anemia and middle-aged healthy males. Thirdly, *TMPRSS6* was associated with serum iron and hemoglobin concentrations in several GWASs for Europeans [15], [35], [36]. The association of *TMPRSS6* with serum ferritin was confirmed in Chinese [14], so was its association with hemoglobin, iron and transferrin saturation concentrations [34]. However, in a GWAS for Australian twin families, *TMPRSS6* was associated with serum ferritin with a borderline *P* value around 10^−4^ [15].

The limitations of our study should be evaluated objectively. Firstly, the sample size is probably not sufficient enough to make some genuine SNPs reach the GWAS significant level. Further study in Chinese with a larger sample size or meta-analysis of GWASs in different populations is recommended. Secondly, the participants in our study were adult males from general population, which might lead to a relative selection bias. Further study in females or children with AA may enhance our findings.Thirdly, only one SNP in one gene was selected to be further tested, so some genuine genes in the potential pathways may be neglected. Further well-designed biological experiments combined with bioinformatic analysis were recommended, especially the quantitative real-time polymerase chain reaction (qRT-PCR). Last but not the least, the role of environmental backgrounds or gene-environment interactions cannot be ignored either.

## Conclusions

In summary, to our knowledge, we are the first to perform the two-stage GWAS in Chinese male population to explore the genetic influence on serum ferritin concentrations. Our study observed that the rs5742933located in *PMS1* was the most significant SNP associated with serum ferritin, which suggested that *PMS1* may be a susceptibility gene affecting serum ferritin concentrations in Chinese male population. The candidate gene *SLC40A1* were closed to gene *PMS1*, so the underlying relation between the *SLC40A1* and *PMS1* needs further study. Our results may partly contribute some evidence to the genetic control of serum ferritin or iron status.
